# Smoking Behavior Change and the Risk of Heart Failure in Patients With Type 2 Diabetes: Nationwide Retrospective Cohort Study

**DOI:** 10.2196/46450

**Published:** 2024-01-10

**Authors:** Jung Eun Yoo, Su-Min Jeong, Kyu Na Lee, Heesun Lee, Ji Won Yoon, Kyungdo Han, Dong Wook Shin

**Affiliations:** 1 Department of Family Medicine Healthcare System Gangnam Center Seoul National University Hospital Seoul Republic of Korea; 2 Department of Medicine Seoul National University College of Medicine Seoul Republic of Korea; 3 Department of Statistics and Actuarial Science Soongsil University Seoul Republic of Korea; 4 Division of Cardiology, Department of Internal Medicine Healthcare System Gangnam Center Seoul National University Hospital Seoul Republic of Korea; 5 Division of Endocrinology and Metabolism, Department of Internal Medicine Healthcare System Gangnam Center Seoul National University Hospital Seoul Republic of Korea; 6 Department of Family Medicine Samsung Medical Center Sungkyunkwan University School of Medicine Seoul Republic of Korea; 7 Supportive Care Center Samsung Medical Center Sungkyunkwan University School of Medicine Seoul Republic of Korea; 8 Department of Clinical Research Design & Evaluation Samsung Advanced Institute for Health Science & Technology Sungkyunkwan University Seoul Republic of Korea

**Keywords:** smoking, change in smoking behavior, cessation, heart failure, type 2 diabetes, diabetes, cardiovascular disease, smoking cessation, smoker, risk factor

## Abstract

**Background:**

Heart failure (HF) is one of the most common initial manifestations of cardiovascular disease in patients with type 2 diabetes. Although smoking is an independent risk factor for HF, there is a lack of data for the incidence of HF according to changes in smoking behaviors in patients with type 2 diabetes.

**Objective:**

We aimed to examine the association between interval changes in smoking behavior and the risk of HF among patients with type 2 diabetes.

**Methods:**

We conducted a retrospective cohort study using the National Health Insurance Service database. We identified 365,352 current smokers with type 2 diabetes who had 2 consecutive health screenings (2009-2012) and followed them until December 31, 2018, for the incident HF. Based on smoking behavior changes between 2 consecutive health screenings, participants were categorized into quitter, reducer I (≥50% reduction) and II (<50% reduction), sustainer (reference group), and increaser groups.

**Results:**

During a median follow-up of 5.1 (IQR 4.0-6.1) years, there were 13,879 HF cases (7.8 per 1000 person-years). Compared to sustainers, smoking cessation was associated with lower risks of HF (adjusted hazard ratio [aHR] 0.90, 95% CI0.86-0.95), whereas increasers showed higher risks of HF than sustainers; heavy smokers who increased their level of smoking had a higher risk of HF (aHR 1.13, 95% CI 1.04-1.24). In the case of reducers, the risk of HF was not reduced but rather increased slightly (reducer I: aHR 1.14, 95% CI 1.08-1.21; reducer II: aHR 1.03, 95% CI 0.98-1.09). Consistent results were noted for subgroup analyses including type 2 diabetes severity, age, and sex.

**Conclusions:**

Smoking cessation was associated with a lower risk of HF among patients with type 2 diabetes, while increasing smoking amount was associated with a higher risk for HF than in those sustaining their smoking amount. There was no benefit from reduction in smoking amount.

## Introduction

### Background

We are currently in a global type 2 diabetes public health crisis; an estimated 426 million individuals have type 2 diabetes [1]. Patients with type 2 diabetes are at increased risk of cardiovascular disease, and heart failure (HF) is one of the most common initial manifestations of cardiovascular disease in patients with type 2 diabetes [2]. Furthermore, type 2 diabetes contributes greatly to the increased morbidity and mortality associated with HF [3]. Thus, identifying modifiable risk factors for HF is critical in this population at high risk of type 2 diabetes.

Smoking is the leading predictor of death in patients with type 2 diabetes [4] and also an independent risk factor for HF and contributes to coronary heart disease, a major cause of HF [5-7]. In particular, smoking has been linked with several factors associated with left ventricular hypertrophy and cardiac dysfunction [8,9]. In addition, recent studies have found that individuals who smoke have at least twice the risk of developing HF than those who never smoke [8,10,11]. The risk is almost 4-fold among smokers who smoke ≥25 cigarettes per day [10].

Limited evidence is currently available, demonstrating that smoking cessation is associated with a reduced risk of incident HF and adverse events related to HF [5]. Some studies have reported that the risks of HF decreased over time after smoking cessation [11,12]. Those who ceased smoking for over 30 years experienced a similar HF risk to never smokers [13]. Former smokers also had similar cardiac structure and function as measured by echocardiography when compared with never smokers [9].

Given the increased risk for HF posed by type 2 diabetes, defining the effect of smoking cessation for HF in a type 2 diabetes cohort is important. Indeed, smoking and type 2 diabetes interrelate in the generation of cardiovascular events [14]. However, most studies have not focused on this high-risk population, and the HF incidence associations between current smoking and smoking cessation with type 2 diabetes are still unknown. Indeed, although smoking behavior can change over time, previous studies measured the association at only 1 timepoint [8-12,15,16], and there is a lack of data for the incidence of HF according to changes in smoking frequency among current smokers. One additional limitation of the previous studies is that the study population was comprised of only older individuals (aged ≥65 years [11]). Another limitation was the small sample sizes and the small number of outcomes of interest. The largest previous study had 3874 HF events among 188,167 individuals [10].

### Study Design and Aim

To fill this knowledge gap, we conducted a nationwide cohort study to investigate whether changes in smoking behavior among patients with type 2 diabetes resulted in subsequent altered risk of HF. By measuring smoking behavior repetitively, we were able to investigate the effects of reduction, increase, and cessation of smoking.

## Methods

### Study Setting

We used a database provided by the National Health Insurance Service (NHIS), a single insurer in Korea [17]. The NHIS provides a mandatory universal insurance system that covers ~97% (about 51 million) of the Korean population and medical aid to ~3% (about 1.5 million) of the population in the lowest income bracket. NHIS provides a free biennial cardiovascular health screening for all insured individuals. Hence, the NHIS retains an extensive data set of the entire Korean population that includes demographics; medical treatment and procedures; disease diagnosis according to the International Classification of Diseases, 10th revision (ICD-10) codes; and the results of health screenings consisting of a self-questionnaire on health behavior, anthropometric measurements, and laboratory test results.

### Study Population

**Figure 1:**
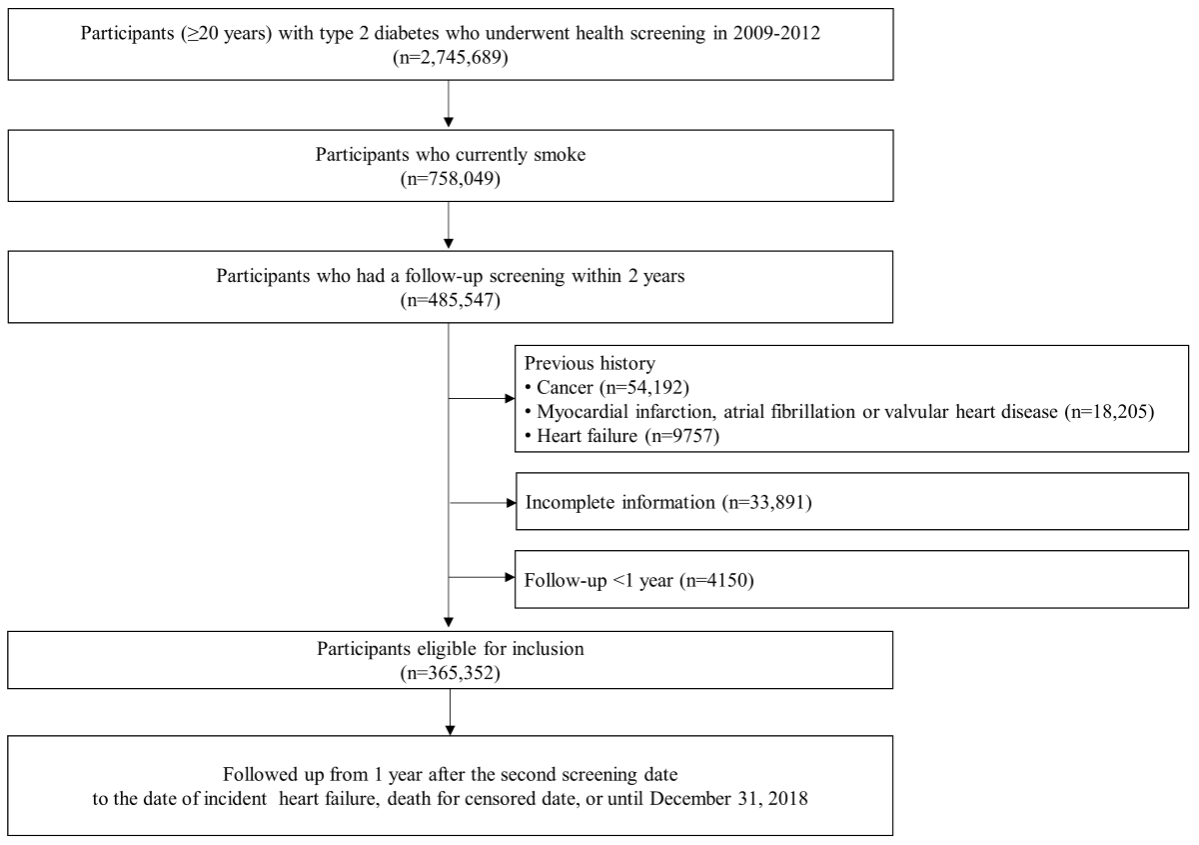
Flowchart for participant recruitment.

Among individuals who underwent a health screening in 2009-2012, we identified 2,745,689 individuals with type 2 diabetes: (1) those with a previous history of type 2 diabetes which was defined as ICD-10 code (E11-14) diagnosis with at least 1 claim for prescription of antidiabetic agents before the health screening or (2) those with fasting plasma glucose 126 mg/dL at the health screening. This definition was based on the consensus of relevant findings widely used in previous studies [18,19]. We then selected current smokers (n=758,049) according to the World Health Organization definition of current smoker [20]. Among these, 485,547 underwent a follow-up health examination within 2 years. We excluded those who had previously been diagnosed with (1) any cancer (n=54,192); (2) myocardial infarction, atrial fibrillation, or valvular heart diseases (n=18,205); or (3) HF (n=9757) before the second health screening. Those with at least 1 missing variable used in the study were also excluded (n=33,891). To reduce the effect of reverse causality, we applied a 1-year lag time by excluding participants who were diagnosed with HF (n=2463) and who died (n=1687) within 1 year after the second health screening. A total of 365,352 participants were included in the primary analysis (Figure 1).

### Ethical Considerations

This study was approved by the institutional review board of Samsung Medical Center (IRB SMC 2022-07-072). The review board waived the requirement for written informed consent from the patients because the data are public and anonymized under confidentiality guidelines.

### Definition of Change in Amount of Smoking Cigarettes

Information on smoking status was obtained from a self-administered questionnaire completed as a part of the NHIS health screening. The participants who identified themselves as current smokers were asked to provide their daily smoking amount (average number of cigarettes per day) and smoking duration in years. According to the number of cigarettes per day at the time of the first screening, participants were categorized into three groups: (1) light smokers (<10 cigarettes per day), (2) moderate smokers (10-19 cigarettes per day), and (3) heavy smokers (≥20 cigarettes per day). Each of the 3 groups was then divided into 5 subgroups by comparing the number of cigarettes per day between the first screening and the follow-up screening. The subgroups were (1) quitters (those who completely ceased smoking), (2) reducers I (those who reduced the number of cigarettes by 50% or more), (3) reducers II (those who reduced the number of cigarettes by 20% or more and less than 50%), (4) sustainers (those who reduced the number of cigarettes by less than 20% or increased by less than 20%), and (5) increasers (those who increased the number of cigarettes by 20% or more).

### Outcome: Ascertainment of HF and Follow-Up

The end point of the study was a newly diagnosed case of HF. HF was defined as the first hospitalization under a primary diagnosis of ICD-10 code I50 as used in previous studies [21-23]. The study participants were followed up from 1 year after the second screening date to the date of incident HF, death or outmigration, or the end of the study period (December 31, 2018), whichever came first.

### Covariates

We considered socioeconomic position as a potential covariate including income level and place of residence. Household income was categorized into quartiles based on insurance premium levels with those covered by Medical Aid being merged into the lowest income quartile. Alcohol consumption was classified as none, mild (<15 g of alcohol per day), moderate (15-30 g of alcohol per day), and heavy (≥30 g of alcohol per day). Regular exercise was defined as >30 minutes of moderate physical activity at least 5 times per week or >20 minutes of strenuous physical activity at least 3 times per week [24]. BMI was calculated as subject weight (kg) divided by square of height (m^2^).

Hypertension was defined as a history of a claim (I10-13 or 15 codes) and antihypertensive medication or systolic blood pressure ≥140 mm Hg or diastolic blood pressure ≥90 mm Hg. Dyslipidemia was defined as a history of a claim with E78 codes and lipid-lowering medications, or total cholesterol level ≥240 mg/dL. Chronic kidney disease was defined as a glomerular filtration rate <60 mL/minute per 1.73 m^2^ as estimated by the Modification of Diet in Renal Disease equation. Chronic obstructive pulmonary disease was defined as a claim with J41-44 codes, and stroke was defined as a claim with codes I63-64.

### Statistical Analysis

Cox proportional hazards regression analysis was performed to evaluate the association between smoking behavior change and incident HF. Model 1 was unadjusted. Model 2 was adjusted for age, sex, income, area of residence, alcohol consumption, duration of smoking, physical activity, BMI, and comorbidities (hypertension, dyslipidemia, chronic kidney disease, chronic obstructive pulmonary disease, and stroke). Model 3 was further adjusted for fasting glucose level, duration of type 2 diabetes, and insulin use. Stratification analyses by smoking levels at the first examination (light, moderate, and heavy smoker), type 2 diabetes severity (duration of type 2 diabetes: new-onset, <5 years, and 5 years, the number of oral antidiabetic agents taken: 0, 1-2, and 3, and use of insulin), age (20-64 years and 65 years), and sex were performed to assess the association of change in smoking behavior with incidence of HF. Statistical analyses were performed using SAS (version 9.4; SAS Institute Inc), and a *P* value <.05 was considered statistically significant.

## Results

### Baseline Characteristics of the Study Population

Table 1 shows the baseline characteristics according to the change in smoking behavior. During the 2 years before initiation of the study, 44.5% (n=162,645) of current smokers sustained their smoking amount, 18.6% (n=67,843) became quitters, 20.7% (n=75,615) reduced their smoking amount (n=27,987, 7.7% and n=47,628, 13% in reducer I and reducer II groups, respectively), and the remaining 16.2% (n=59,249) increased their smoking amount. Compared to sustainers, quitters tended to be older, women, nondrinkers, and light smokers, engage in more regular exercise, have more comorbid conditions and longer duration of type 2 diabetes, and use multiple oral antidiabetic agents and insulin.

**Table 1:** Baseline characteristics of the study population according to smoking behavior change (2009-2011).

Variables								Total (N=365,352)				Smoking behavior change																
												Quitter (n=67,843)						Reducer I (n=27,987)		Reducer II (n=47,628)				Sustainer (n=162,645)			Increaser (n=59,249)	
Age (years), mean (SD)								52.1 (11.1)				54.6 (11.2)						53.5 (11.6)		51.1 (11.0)				51.5 (10.7)			51.2 (11.2)	
Sex (male), n (%)								347,685 (95.2)				61,788 (91.1)						26,216 (93.7)		45,969 (96.5)				157,461 (96.8)			56,251 (94.9)	
**Income, n (%)**																												
						Q1 (lowest)	67,489 (18.5)				12,534 (18.5)						5954 (21.3)		8530 (17.9)				29,205 (18)			11,266 (19)		
						Q2	70,644 (19.3)				12,925 (19.1)						5776 (20.6)		9184 (19.3)				30,797 (18.9)			11,962 (20.2)		
						Q3	107,573 (29.4)				18,473 (27.2)						7890 (28.2)		14,295 (30)				49,074 (30.2)			17,841 (30.1)		
						Q4 (highest)	119,646 (32.8)				23,911 (35.2)						8367 (29.9)		15,619 (32.7)				53,569 (32.9)			18,180 (30.7)		
Place of residence (urban), n (%)								162,216 (44.4)				29,942 (44.1)						12,324 (44)		21,310 (44.7)				72,574 (44.6)			26,066 (44)	
**Alcohol consumption, n (%)**																												
					None		104,751 (28.7)				28,335 (41.8)						8453 (30.2)		12,089 (25.4)				40,614 (25.0)			15,260 (25.8)		
					Mild		115,893 (31.7)				20,399 (30.1)						10,357 (37)		16,293 (34.2)				50,944 (31.3)			17,900 (30.2)		
					Moderate		79,341 (21.7)				10,789 (15.9)						5538 (19.8)		10,927 (22.9)				38,817 (23.9)			13,270 (22.4)		
					Heavy		65,367 (17.9)				8320 (12.3)						3639 (13)		8319 (17.5)				32,270 (19.8)			12,819 (21.6)		
**Smoking status^a^ (CPD^b^), n (%)**																												
				Light (<10)				34,791 (9.5)				12,376 (18.2)						1122 (4)		2536 (5.3)				5905 (3.6)			12,852 (21.7)	
				Moderate (10-19)				132,370 (36.2)				26,278 (38.7)						5605 (20)		15,609 (32.8)				51,907 (31.9)			32,971 (55.7)	
				Heavy (≥20)				198,191 (54.3)				29,189 (43.0)						21,260 (76)		29,483 (61.9)				104,833 (64.5)			13,426 (22.7)	
**Duration of smoking (years)^a^, n (%)**																												
			<5				10,473 (2.9)				3744 (5.5)						690 (2.5)		877 (1.8)				2862 (1.8)			2300 (3.9)		
			5-9				14,065 (3.9)				3595 (5.3)						1127 (4)		1535 (3.2)				4926 (3)			2882 (4.9)		
			10-19				82,421 (22.6)				14,547 (21.4)						5852 (20.9)		10,968 (23)				35,743 (22)			15,311 (25.8)		
			20-29				119,500 (32.7)				19,655 (29)						8439 (30.2)		16,132 (33.9)				56,215 (34.6)			19,059 (32.2)		
			≥30				138,893 (38)				26,302 (38.8)						11,879 (42.4)		18,116 (38)				62,899 (38.7)			19,697 (33.2)		
**Pack-years of smoking^a^, n (%)**																												
		<10					77,059 (21.1)				20,834 (30.7)						3844 (13.7)		6727 (14.1)				24,107 (14.8)			21,547 (36.4)		
		10-20					100,327 (27.5)				17,776 (26.2)						6116 (21.9)		12,194 (25.6)				43,776 (26.9)			20,465 (34.5)		
		20-30					82,568 (22.6)				12,799 (18.9)						6337 (22.6)		10,510 (22.1)				43,534 (26.8)			9388 (15.8)		
		≥30					105,398 (28.9)				16,434 (24.2)						11,690 (41.8)		18,197 (38.2)				51,228 (31.5)			7849 (13.3)		
**Physical activity**																												
		No, n (%)					289,791 (79.3)				51,429 (75.8)						21,728 (77.6)		37,770 (79.3)				130,923 (80.5)			47,941 (80.9)		
		Irregular, n (%)					55,630 (15.2)				11,881 (17.5)						4535 (16.2)		7422 (15.6)				23,451 (14.4)			8341 (14.1)		
		Regular, n (%)					19,931 (5.5)				4533 (6.7)						1724 (6.16)		2436 (5.1)				8271 (5.1)			2967 (5)		
		BMI (kg/m^2^), mean (SD)					24.8 (3.3)				25.0 (3.2)						24.7 (3.3)		24.8 (3.4)				24.7 (3.3)			24.8 (3.4)		
		Systolic blood pressure (mm Hg), mean (SD)					126.8 (14.6)				127.3 (14.6)						127.0 (14.9)		126.7 (14.4)				126.7 (14.5)			126.7 (14.7)		
		Diastolic blood pressure (mm Hg), mean (SD)					78.9 (9.9)				78.8 (9.8)						78.8 (10.0)		79.0 (9.9)				79.0 (9.9)			79.0 (10.0)		
		Fasting glucose (mg/dL), mean (SD)					137.8 (51.8)				138.4 (50.8)						137.7 (52.4)		136.6 (50.8)				137.4 (51.5)			139.3 (54.1)		
		Total cholesterol (mg/dL), mean (SD)					193.4 (43.2)				192.0 (46.3)						192.1 (43.7)		193.5 (41.7)				194.1 (42.9)			193.7 (41.6)		
		Estimated glomerular filtration rate, mean (SD)					92.5 (48.3)				90.2 (48.2)						91.7 (45.3)		92.9 (47.5)				93.1 (48.8)			93.4 (48.7)		
**Comorbidity, n (%)**																												
	Hypertension							169,279 (46.3)				34,373 (50.7)						13,534 (48.4)		21,385 (44.9)				73,440 (45.2)			26,547 (44.8)	
	Dyslipidemia							138,938 (38)				28,440 (41.9)						10,645 (38)		17,725 (37.2)				60,174 (37)			21,954 (37.1)	
	Chronic kidney disease							17,653 (4.8)				4470 (6.6)						1632 (5.8)		2127 (4.5)				6815 (4.2)			2609 (4.4)	
	COPD^c^							94,232 (25.8)				19,870 (29.3)						7745 (27.7)		11,607 (24.4)				40,107 (24.7)			14,903 (25.2)	
	Stroke							21,042 (5.8)				5476 (8.1)						1933 (6.9)		2339 (4.9)				8057 (5.0)			3237 (5.5)	
**Duration of type 2 diabetes, n (%)**																												
		New-onset							203,127 (55.6)				33,965 (50.1)		14,948 (53.4)						27,553 (57.9)			93,270 (57.4)			33,391 (56.4)	
		<5 years							84,232 (23.1)				16,624 (24.5)		6661 (23.8)						10,512 (22.1)			36,694 (22.6)			13,741 (23.2)	
		≥5 years							77,993 (21.4)				17,254 (25.4)		6378 (22.8)						9563 (20.1)			32,681 (20.1)			12,117 (20.5)	
**Oral antidiabetic agents, n (%)**																												
		0							176,620 (48.3)				27,853 (41.1)		12,977 (46.4)						24,259 (50.9)			81,937 (50.4)			29,594 (50)	
		1-2							132,677 (36.3)				27,780 (41)		10,467 (37.4)						16,557 (34.8)			57,123 (35.1)			20,750 (35)	
		≥3							56,055 (15.3)				12,210 (18)		4543 (16.2)						6812 (14.3)			23,585 (14.5)			8905 (15)	
Use of insulin, n (%)										19,722 (5.4)				4882 (7.2)		1670 (6)						2338 (4.9)			7765 (4.8)			3067 (5.2)

^a^Information related to smoking status is based on data from the first screening period (2009).

^b^CPD: cigarettes per day.

^c^COPD: chronic obstructive pulmonary disease.

### Change in Smoking Behavior and HF

During a median follow-up of 5.1 (IQR 4.0-6.1) years, there were 13,879 HF cases (7.8 per 1000 person-years). Table 2 shows the associations between change in smoking behavior and the risk of HF, with sustainers as a reference group.

There was no overall difference in HF risk between increasers and sustainers (adjusted hazard ratio [aHR] 1.02, 95% CI 0.97-1.07), but heavy smokers who increased their level of smoking had a 13% higher HF risk (aHR 1.13, 95% CI 1.04-1.24). In the case of reducers, the risk of HF was not reduced but rather increased slightly (aHR 1.14, 95% CI 1.08-1.21, for reducer I; aHR 1.03, 95% CI 0.98-1.09, for reducer II). Conversely, quitters had a lower risk of incident HF after adjusting for confounding factors including type 2 diabetes severity (aHR 0.90, 95% CI 0.86-0.95). When stratified by smoking level at the first screening, moderate-to-heavy smokers who became quitters had a lower risk of HF than sustainers (aHR 0.90, 95% CI 0.83-0.98 for moderate smoker; aHR 0.89, 95% CI 0.83-0.94 for heavy smoker).

**Table 2:** Associations of smoking behavior change (2009-2011) with heart failure during 5.1 years of follow-up (N=365,352).

Smoking behavior change 2009-2011^a^			Participants, n (%)	Events, n (%)	Duration (person-years)	IR^b^	Model 1^c^ HR^d^ (95% CI)	Model 2^e^ HR (95% CI)	Model 3^f^ HR (95% CI)
**All current smoker**									
	Quitter		67,843 (18.6)	2850 (20.5)	333,223.1	8.6	1.17 (1.12-1.23)	0.92 (0.88-0.96)	0.90 (0.86-0.95)
	Reducer I		27,987 (7.7)	1361 (9.8)	136,019.6	10.0	1.38 (1.30-1.46)	1.15 (1.09-1.22)	1.14 (1.08-1.21)
	Reducer II		47,628 (13.0)	1726 (12.4)	234,148.6	7.4	1.01 (0.96-1.07)	1.03 (0.98-1.09)	1.03 (0.98-1.09)
	Sustainer		162,645 (44.5)	5779 (41.6)	796,568.8	7.3	1 (Reference)	1 (Reference)	1 (Reference)
	Increaser		59,249 (16.2)	2163 (15.6)	288,377.6	7.5	1.04 (0.99-1.09)	1.03 (0.98-1.08)	1.02 (0.97-1.07)
*P* value			N/A^g^	N/A	N/A	N/A	<.001	<.001	<.001
**Smoking status in 2009^h^**									
	**Light smoker (n=34,791)**								
		Quitter	12,376 (3.4)	585 (4.2)	59,050.7	9.9	1.23 (1.05-1.43)	1.13 (0.97-1.32)	1.12 (0.96-1.30)
		Reducer I	1122 (0.3)	41 (0.3)	5350.9	7.7	0.95 (0.68-1.33)	0.89 (0.64-1.24)	0.89 (0.64-1.24)
		Reducer II	2536 (0.7)	155 (1.1)	11,967.5	13.0	1.61 (1.31-1.97)	1.43 (1.16-1.75)	1.45 (1.18-1.78)
		Sustainer	5905 (1.6)	227 (1.6)	28,215.5	8.0	1 (Reference)	1 (Reference)	1 (Reference)
		Increaser	12,852 (3.5)	534 (3.8)	61,458.2	8.7	1.08 (0.92-1.26)	1.16 (0.99-1.35)	1.16 (0.99-1.35)
	**Moderate smoker (n=132,370)**								
		Quitter	26,278 (7.2)	1020 (7.3)	128,926.6	7.9	1.18 (1.10-1.28)	0.93 (0.86-1.01)	0.90 (0.83-0.98)
		Reducer I	5605 (1.5)	233 (1.7)	27,180.4	8.6	1.29 (1.13-1.48)	0.97 (0.84-1.11)	0.96 (0.84-1.10)
		Reducer II	15,609 (4.3)	536 (3.9)	76,381.6	7.0	1.06 (0.96-1.16)	0.99 (0.90-1.09)	0.98 (0.89-1.08)
		Sustainer	51,907 (14.2)	1666 (12.0)	252,254.2	6.6	1 (Reference)	1 (Reference)	1 (Reference)
		Increaser	32,971 (9.0)	1082 (7.8)	160,352	6.7	1.02 (0.95-1.10)	1.05 (0.97-1.13)	1.03 (0.95-1.11)
	**Heavy smoker (n=198,191)**								
		Quitter	29,189 (8.0)	1245 (9.0)	145,245.8	8.6	1.13 (1.06-1.20)	0.90 (0.85-0.96)	0.89 (0.83-0.94)
		Reducer I	21,260 (5.8)	1087 (7.8)	103,488.2	10.5	1.40 (1.31-1.50)	1.19 (1.12-1.28)	1.19 (1.11-1.28)
		Reducer II	29,483 (8.1)	1035 (7.5)	145,799.5	7.1	0.94 (0.88-1.01)	1.03 (0.96-1.10)	1.03 (0.96-1.10)
		Sustainer	104,833 (28.7)	3886 (28.0)	516,099.2	7.5	1 (Reference)	1 (Reference)	1 (Reference)
		Increaser	13,426 (3.7)	547 (3.9)	66,567.3	8.2	1.09 (0.99-1.19)	1.17 (1.07-1.28)	1.13 (1.04-1.24)
*P* for interaction			N/A	N/A	N/A	N/A	<.001	<.001	<.001

^a^Quitters are those who ceased smoking; reducer I are those who reduced the number of cigarettes by 50% or more; reducer II are those who reduced the number of cigarettes by 20% or more and by less than 50%; sustainers are those who reduced the number of cigarettes by less than 20% or increased by less than 20%; increasers are those who increased the number of cigarettes by 20% or more.

^b^IR: incidence rate per 1000 person-years.

^c^Model 1: unadjusted.

^d^HR: hazard ratio.

^e^Model 2: adjusted for age, sex, socioeconomic position (income level and place of residence), alcohol consumption, duration of smoking, physical activity, BMI, and comorbidities (hypertension, dyslipidemia, chronic kidney disease, chronic obstructive pulmonary disease, and stroke).

^f^Model 3: Model 2 + adjusted for fasting glucose level, duration of type 2 diabetes, and use of insulin.

^g^N/A: not applicable.

^h^Light smoker: <10 cigarettes per day; moderate smokers: 10-19 cigarettes per day; heavy smokers: ≥20 cigarettes per day.

### Stratified Analysis

**Figure 2:**
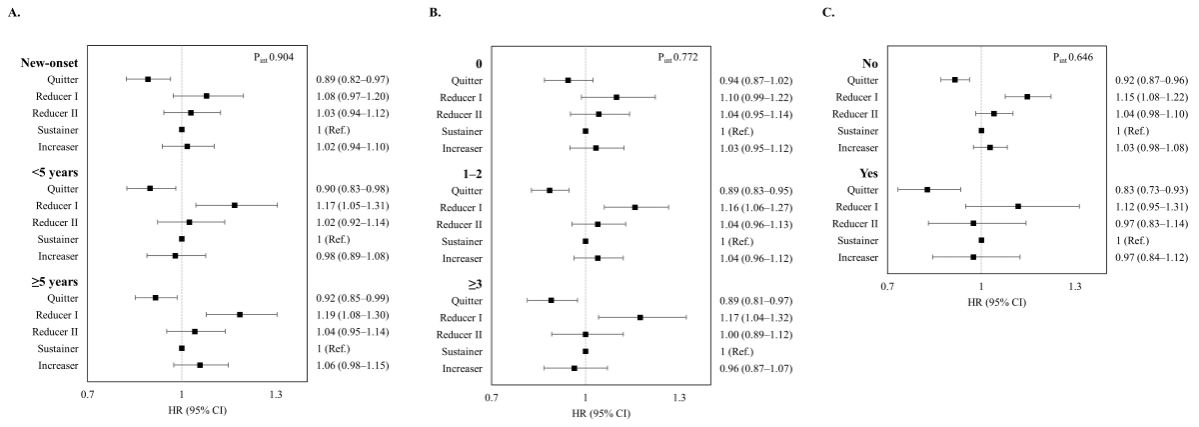
Association of smoking behavior change (2009-2012) with the incidence of heart failure stratified by (A) duration of type 2 diabetes, (B) number of oral antidiabetic agents, and (C) use of insulin. HRs are adjusted for age, sex, socioeconomic position (income level and place of residence), alcohol consumption, duration of smoking, physical activity, BMI, comorbidities (hypertension, dyslipidemia, chronic kidney disease, chronic obstructive pulmonary disease, and stroke), fasting glucose level, duration of type 2 diabetes, and use of insulin. HR: hazard ratio; quitter: those who ceased smoking; reducer I: those who reduced the number of cigarettes by 50% or more; reducer II: those who reduced the number of cigarettes by 20% or more and by less than 50%; sustainer: those who reduced the number of cigarettes by less than 20% or increased by less than 20%; increaser: those who increased the number of cigarettes by 20% or more.

Figure 2 presents the associations between smoking behavior change and risk of HF incidence according to type 2 diabetes severity. Smoking cessation remained predictive of a lower incidence of HF, regardless of the duration of type 2 diabetes, number of oral antidiabetic agents, and use of insulin. In stratified analyses according to age and sex, the results were also consistent with the main findings (Table 3).

**Table 3:** Stratified analysis on the associations of smoking behavior change (2009-2011)^a^ and the risk of heart failure.

Smoking behavior in 2009-2011				Participants, n (%)		Events, n (%)		Duration (Person-years)		IR^b^		Model 1^c^ HR^d^ (95% CI)		Model 2^e^ HR (95% CI)		Model 3^f^ HR (95% CI)
**Age**																
	**20-64 years**															
		Quitter	55,007 (15.1)		1594 (11.5)		272,553.4		5.8		1.04 (0.98-1.10)		0.92 (0.87-0.98)		0.91 (0.85-0.96)	
		Reducer I	23,080 (6.3)		783 (5.6)		113,242.4		6.9		1.24 (1.15-1.34)		1.16 (1.08-1.25)		1.15 (1.06-1.24)	
		Reducer II	42,174 (11.5)		1156 (8.3)		208,554.5		5.5		0.99 (0.93-1.06)		1.02 (0.95-1.09)		1.02 (0.96-1.09)	
		Sustainer	144,252 (39.5)		3957 (28.5)		709,688.7		5.6		1 (Reference)		1 (Reference)		1 (Reference)	
		Increaser	52,126 (14.3)		1443 (10.4)		255,321.3		5.7		1.02 (0.96-1.08)		1.04 (0.98-1.10)		1.03 (0.97-1.09)	
	≥**65 years**															
		Quitter	12,836 (3.5)		1256 (9.0)		60,669.8		20.7		0.99 (0.92-1.06)		0.92 (0.85-0.99)		0.90 (0.84-0.97)	
		Reducer I	4907 (1.3)		578 (4.2)		22,777.2		25.4		1.22 (1.11-1.34)		1.14 (1.04-1.25)		1.14 (1.03-1.25)	
		Reducer II	5454 (1.5)		570 (4.1)		25,594.1		22.3		1.06 (0.97-1.17)		1.06 (0.96-1.16)		1.06 (0.96-1.16)	
		Sustainer	18,393 (5.0)		1822 (13.1)		86,880.2		21.0		1 (Reference)		1 (Reference)		1 (Reference)	
		Increaser	7123 (1.9)		720 (5.2)		33,056.3		21.8		1.05 (0.96-1.14)		1.01 (0.93-1.10)		1.01 (0.92-1.10)	
*P* value for interaction				N/A^g^		N/A		N/A		N/A		.33		.90		.93
**Sex**																
	**Men**															
		Quitter	61,788 (16.9)		2479 (17.9)		304,628.3		8.1		1.14 (1.08-1.19)		0.91 (0.87-0.95)		0.89 (0.85-0.94)	
		Reducer I	26,216 (7.2)		1245 (9.0)		127,646.9		9.8		1.37 (1.29-1.46)		1.16 (1.09-1.24)		1.16 (1.09-1.23)	
		Reducer II	45,969 (12.6)		1632 (11.8)		226,394.6		7.2		1.01 (0.96-1.07)		1.03 (0.98-1.09)		1.04 (0.98-1.10)	
		Sustainer	157,461 (43.1)		5487 (39.5)		772,035.7		7.1		1 (Reference)		1 (Reference)		1 (Reference)	
		Increaser	56,251 (15.4)		1997 (14.4)		274,163.9		7.3		1.03 (0.98-1.08)		1.04 (0.99-1.09)		1.03 (0.98-1.08)	
	**Women**															
		Quitter	6055 (1.7)		371 (2.7)		28,594.9		13.0		1.09 (0.93-1.27)		0.98 (0.84-1.14)		0.97 (0.83-1.13)	
		Reducer I	1771 (0.5)		116 (0.8)		8372.7		13.9		1.16 (0.94-1.44)		1.03 (0.83-1.27)		1.04 (0.84-1.29)	
		Reducer II	1659 (0.5)		94 (0.7)		7754.0		12.1		1.02 (0.81-1.29)		0.97 (0.77-1.22)		0.97 (0.77-1.22)	
		Sustainer	5184 (1.4)		292 (2.1)		24,533.2		11.9		1 (Reference)		1 (Reference)		1 (Reference)	
		Increaser	2998 (0.8)		166 (1.2)		14,213.7		11.7		0.98 (0.81-1.19)		0.94 (0.78-1.14)		0.95 (0.79-1.15)	
*P* value for interaction				N/A		N/A		N/A		N/A		.67		.26		.30

^a^Quitters are those who quit smoking; reducer I are those who reduced the number of cigarettes by 50% or more; reducer II are those who the number of cigarettes by 20% or more and less than 50%; sustainer are those who reduced the number of cigarettes by less than 20% or increased by less than 20%; increaser are those who increased the number of cigarettes by 20% or more.

^b^IR: incidence rate per 1000 person-years.

^c^Model 1: unadjusted.

^d^HR: hazard ratio.

^e^Mode 2: Adjusted for age, sex, socioeconomic position (income level and place of residence), alcohol consumption, duration of smoking, physical activity, BMI, and comorbidities (hypertension, dyslipidemia, chronic kidney disease, chronic obstructive pulmonary disease, and stroke).

^f^Mode 3: Model 2 + adjusted for fasting glucose level, duration of type 2 diabetes, and use of insulin.

^g^N/A: not applicable.

## Discussion

### Principal Findings

In this large cohort study with repetitive measurements of smoking behavior, we found that increasers, particularly those who were initially heavy smokers, were associated with higher risks of HF than sustainers. In the case of reducers, the incidence of HF was not decreased but slightly increased. Smoking cessation was associated with a decreased risk of incident HF among moderate-to-heavy smokers with type 2 diabetes. Our stratified analyses also demonstrated that smoking cessation was consistently associated with a lower HF risk among type 2 diabetes with various characteristics.

The association between smoking and atherosclerotic coronary artery disease, a major cause of HF [7], is well established. The acute prothrombotic, proadrenergic, and proinflammatory properties of smoking are believed to underlie smoking-associated atherosclerosis and cardiovascular disease [11]. In addition, smoking directly leads to left ventricular hypertrophy. In vitro, nicotine stimulates the proliferation of endothelial cells and vascular smooth muscle cells [25], and chronic inhalation of carbon monoxide induces cardiac hypertrophy [25]. Consistently, we showed that heavy smokers with type 2 diabetes who increased their amount of smoking during 2 consecutive screenings had a higher risk of HF than those who sustained their smoking amount. In type 2 diabetes, hyperglycemia, insulin resistance, and hyperinsulinemia trigger a cascade of deleterious effects that contribute to the development of HF via ischemic cardiomyopathy and diabetic cardiomyopathy [26,27]. There have been many studies showing that the onset and progression of macrovascular and microvascular complications of type 2 diabetes are highly associated with smoking [28]. Therefore, it is possible that smoking and type 2 diabetes interact and further increase the risk of HF.

Harm reduction strategy is aimed at reducing the adverse health effects of smoking by reducing the number of cigarettes smoked each day [29]. However, no evidence that cardiovascular risks are reduced by smoking reduction have been provided [29-31]. Consistently, we found that smoking reduction among patients with type 2 diabetes had no benefit for reducing HF risk. Type 2 diabetes itself is a high-risk condition of HF and also interrelates with smoking in relation to cardiovascular events [14]. Thus, the threshold at which smoking affects the development of HF appears to be low, and smoking reduction may not be sufficient to reduce the risk of HF. Moreover, substantial smoking reduction of ≥50% worsened rather than leaving unchanged HF risk, particularly in heavy smokers. The disease burden attributable to smoking is undoubtedly highest in heavy smokers. For heavy smokers, the effect of smoking may persist after reducing smoking, leading to further deterioration of cardiovascular health. Another possible explanation for this phenomenon is that smoking is primarily a nicotine-seeking behavior, and smokers who reduce tend to compensate by smoking more of each cigarette and by taking more and deeper puffs [29]. This results in a much smaller proportional reduction in nicotine intake (and in the intake of associated tar and other toxins) than the reduction in number of cigarettes suggests [29]. The results of this study support health warnings that there is no safe level of smoking for preventing cardiovascular disease including HF. Smokers with type 2 diabetes need to have a goal of complete smoking cessation in order to lower their risk of HF.

Previous studies have reported that smoking cessation by patients with type 2 diabetes reduces the risk of cardiovascular events compared with those who continue to smoke [32]. Additionally, smoking cessation generally decreases vascular disease risk for patients who are newly diagnosed with type 2 diabetes [33]. Smoking cessation has been shown to reverse smoking-associated endothelial dysfunction, which reduces the cardiovascular disease risk of smoking soon after cessation [6]. Smoking-associated alterations of the myocardium, such as left ventricular remodeling and dysfunction, were also reversible after smoking cessation [9]. We confirmed that smoking cessation, even from heavy smoking, substantially reduced the risk of HF.

It is well known that smoking and type 2 diabetes interrelate. Type 2 diabetes is one of the smoking-related diseases, and smoking cessation reduces the risk of type 2 diabetes [34-36]. In type 2 diabetes, glycemic control tends to worsen after quitting, which can last up to 3 years [37]. Consistently, in this study, analyzing 2-year interval changes, quitters were prescribed more glucose-lowering therapies but did not show a greater decrease in fasting glucose than other groups, which possibly reflects worsened short-term glycemic control in quitters (Table S1 and S2 in Multimedia Appendix 1). Therefore, the reduced risk of HF from smoking cessation is mainly from the effect of smoking cessation per se, and the benefits of smoking cessation may outweigh poorer glycemic control from smoking cessation with respect to HF among patients with type 2 diabetes. If we could track quitters over a longer period, long-term quitters might achieve improved glycemic control.

Smoking cessation consistently decreased the risk of HF even after various stratifications. Interestingly, while the risk of HF among patients with type 2 diabetes usually increases with age and severity or duration of type 2 diabetes, the preventive effect of smoking cessation on HF was also observed among young participants, those with new-onset type 2 diabetes, and those taking fewer oral antidiabetic agents without insulin use. These findings suggest that increased HF risk associated with smoking among patients with type 2 diabetes can be attenuated by smoking cessation.

Current guidelines, such as those of the American Diabetes Association, emphasize that smoking cessation is a major target for the prevention of type 2 diabetes–associated cardiovascular diseases [38]. However, many patients continue to smoke even after a diagnosis of type 2 diabetes [39]. The success rate of smoking cessation among type 2 diabetes is not high, with some studies reporting cessation to be under 20% [40]. In addition to cardiovascular disease, we showed that smoking cessation had a consistent effect on reducing the risk of HF, regardless of type 2 diabetes severity. This study which demonstrates the additional hazards of HF associated with continued smoking and the benefits of smoking cessation among patients with type 2 diabetes provide direct evidence for the need for clinical practitioners to intervene to change smoking behavior.

### Limitations

There are several limitations of our study. First, we defined type 2 diabetes from health claim data not from clinical records and, therefore, could be subject to the risk of under- or overascertainment. However, we used the diagnosis code and medication records together, which have been shown to have high accuracy [41]. Second, since smoking behaviors were based on self-reported questionnaire without using biochemical verification, misclassification from recall or social desirability bias could exist. However, self-reported smoking behavior has been reported to be relatively accurate with 87.5% sensitivity and 89.2% specificity [42]. Third, because we used administrative data, we did not have sufficient clinical information on HF including phenotypes of incident HF, etiology of HF, or plasma brain natriuretic peptide levels.

### Conclusions

In this large population-based cohort study, we demonstrated that increasing smoking amount was associated with a higher risk for HF compared to maintaining smoking amount, while smoking cessation was associated with a lower risk of HF among patients with type 2 diabetes. There was no benefit from the reduction in smoking amount. These findings suggest that smoking cessation should be reinforced to prevent HF in populations at high risk and with type 2 diabetes.

## Appendix

Multimedia Appendix 1 Changes in lifestyle and type 2 diabetes severity according to smoking behavior change (2009 through 2011). Changes in weight and laboratory findings according to smoking behavior change (2009 through 2011).

## Abbreviations

aHR: adjusted hazard ratio
HF: heart failure
ICD-10: International Classification of Diseases, 10th revision
NHIS: National Health Insurance Service

## Data Availability

The data sets generated during and analyzed during this study are not publicly available because restrictions apply to the availability of the data, which were used with permission for the present study. However, they may be available from the corresponding author on reasonable request and with permission from the Korean National Health Insurance Service.
