# Supplementary material for: Smoking Behavior Change and the Risk of Heart Failure in Patients With Type 2 Diabetes: Nationwide Retrospective Cohort Study
Source: JMIR Public Health Surveill. 2024 Jan 10;10:e46450. doi: 10.2196/46450 (PMC10809165; doi:10.2196/46450)
Supplement: Multimedia Appendix 1 [file publichealth_v10i1e46450_app1.docx]

**Multimedia Appendix 1**

**Table S1**. Changes in lifestyle and type 2 diabetes severity according to smoking behavior change (2009 through 2011)

| Variables | | Smoking behavior change^a^ | | | | | | | | |
| --- | --- | --- | --- | --- | --- | --- | --- | --- | --- | --- |
| pre | post | Quitter  (N=67,843) | Reducer I  (N=27,987) | | Reducer II  (N=47,628) | | Sustainer  (N=162,645) | | Increaser  (N=59,249) | |
|  |  |  |  | |  | |  | |  | |
| Alcohol consumption | |  |  | |  | |  | |  | |
| None | None | 15,871 (23.4) | 5,572 (19.9) | | 8,448 (17.7) | | 29,547 (18.2) | | 11,210 (18.9) | |
|  | Mild | 2,733 (4.0) | 1,111 (4.0) | | 1,838 (3.9) | | 6,268 (3.8) | | 2,672 (4.5) | |
|  | Moderate | 501 (0.7) | 261 (0.9) | | 373 (0.8) | | 1,648 (1.0) | | 744 (1.3) | |
|  | Heavy | 338 (0.5) | 139 (0.5) | | 257 (0.5) | | 1,004 (0.6) | | 572 (1.0) | |
| Mild | None | 7,005 (10.3) | 1,845 (6.6) | | 2,497 (5.2) | | 7,669 (4.7) | | 2,931 (5.0) | |
|  | Mild | 11,224 (16.5) | 4,974 (17.8) | | 8,536 (17.9) | | 28,532 (17.5) | | 10,528 (17.8) | |
|  | Moderate | 3,246 (4.8) | 1,292 (4.6) | | 2,604 (5.5) | | 10,783 (6.6) | | 4,768 (8.1) | |
|  | Heavy | 1,184 (1.8) | 453 (1.6) | | 837 (1.8) | | 3,822 (2.4) | | 2,180 (3.7) | |
| Moderate | None | 2,970 (4.4) | 538 (1.9) | | 637 (1.3) | | 1,991 (1.2) | | 666 (1.1) | |
|  | Mild | 4,498 (6.6) | 2,697 (9.6) | | 4,141 (8.7) | | 11,643 (7.2) | | 3,414 (5.8) | |
|  | Moderate | 4,456 (6.5) | 2,180 (7.8) | | 4,555 (9.6) | | 16,814 (10.3) | | 5,243 (8.9) | |
|  | Heavy | 2,384 (3.5) | 860 (3.1) | | 1,886 (4.0) | | 8,827 (5.4) | | 3,846 (6.5) | |
| Heavy | None | 2,489 (3.7) | 498 (1.8) | | 507 (1.1) | | 1,407 (0.8) | | 453 (0.8) | |
|  | Mild | 1,944 (2.9) | 1,575 (5.6) | | 1,778 (3.7) | | 4,501 (2.8) | | 1,286 (2.2) | |
|  | Moderate | 2,586 (3.8) | 1,805 (6.5) | | 3,395 (7.1) | | 9,572 (5.9) | | 2,515 (4.2) | |
|  | Heavy | 4,414 (6.5) | 2,187 (7.8) | | 5,339 (11.2) | | 18,617 (11.5) | | 6,221 (10.5) | |
| Physical activity | |  |  | |  | |  | |  | |
| Non | Non | 43,127 (63.6) | 18,755 (67.0) | | 32,977 (69.2) | | 114,176 (70.2) | | 41,002 (69.2) | |
|  | Irregular | 7,509 (11.1) | 2,969 (10.6) | | 4,872 (10.2) | | 15,101 (9.3) | | 5,225 (8.8) | |
|  | Regular | 2,460 (3.6) | 978 (3.5) | | 1,297 (2.7) | | 4,429 (2.7) | | 1,579 (2.7) | |
| Irregular | Non | 6,349 (9.4) | 2,249 (8.0) | | 3,799 (8.0) | | 13,177 (8.1) | | 5,424 (9.2) | |
|  | Irregular | 3,296 (4.8) | 1,163 (4.2) | | 1,923 (4.0) | | 6,329 (3.9) | | 2,328 (3.9) | |
|  | Regular | 1,217 (1.8) | 418 (1.5) | | 685 (1.4) | | 2,148 (1.3) | | 773 (1.3) | |
| Regular | Non | 1,953 (2.9) | 724 (2.6) | | 994 (2.1) | | 3,570 (2.2) | | 1,515 (2.6) | |
|  | Irregular | 1,076 (1.6) | 403 (1.4) | | 627 (1.3) | | 2,021 (1.2) | | 788 (1.3) | |
|  | Regular | 856 (1.3) | 328 (1.2) | | 454 (1.0) | | 1,694 (1.0) | | 615 (1.0) | |
| Number of oral antidiabetic agents | | | |  | |  | |  | |  |
| 0 | 0 | 25,391 (37.4) | 11,908 (42.6) | | 22,509 (47.3) | | 76,202 (46.9) | | 27,419 (46.3) | |
|  | 1–2 | 6,315 (9.3) | 2,327 (8.3) | | 3,852 (8.1) | | 13,544 (8.3) | | 4,846 (8.2) | |
|  | ≥3 | 1,235 (1.8) | 460 (1.6) | | 703 (1.5) | | 2,453 (1.5) | | 962 (1.6) | |
| 1–2 | 0 | 2,205 (3.2) | 972 (3.5) | | 1,544 (3.2) | | 5,166 (3.2) | | 1,950 (3.3) | |
|  | 1–2 | 18,638 (27.5) | 6,967 (24.9) | | 10,992 (23.1) | | 37,763 (23.2) | | 13,635 (23.0) | |
|  | ≥3 | 5,444 (8.0) | 1,924 (6.9) | | 2,966 (6.2) | | 9,976 (6.1) | | 3,813 (6.4) | |
| ≥3 | 0 | 257 (0.4) | 97 (0.4) | | 206 (0.4) | | 569 (0.4) | | 225 (0.4) | |
|  | 1–2 | 2,827 (4.2) | 1,173 (4.2) | | 1,713 (3.6) | | 5,816 (3.6) | | 2,269 (3.8) | |
|  | ≥3 | 5,531 (8.2) | 2,159 (7.7) | | 3,143 (6.6) | | 11,156 (6.9) | | 4,130 (7.0) | |
| Use of insulin | |  |  | |  | |  | |  | |
| No | No | 61,244 (90.3) | 25,619 (91.5) | | 44,394 (93.2) | | 151,822 (93.4) | | 54,868 (92.6) | |
|  | Yes | 2,801 (4.1) | 942 (3.4) | | 1,298 (2.7) | | 4,222 (2.6) | | 1,667 (2.8) | |
| Yes | No | 1,717 (2.5) | 698 (2.5) | | 896 (1.9) | | 3,058 (1.8) | | 1,314 (2.2) | |
|  | Yes | 2,081 (3.1) | 728 (2.6) | | 1,040 (2.2) | | 3,543 (2.2) | | 1,400 (2.4) | |

Data are presented as number (%).

P value <0.001 for all.

^a^ Quitter, those who ceased smoking; Reducer I, those who reduced the number of cigarettes by 50% or more; Reducer II, those who reduced the number of cigarettes by 20% or more and by less than 50%; Sustainer, those who reduced the number of cigarettes by less than 20% or increased by less than 20%; Increaser, those who increased the number of cigarettes by 20% or more.

**Table S2**. Changes in weight and laboratory findings according to smoking behavior change (2009 through 2011)

| Variables | Smoking behavior change | | | | |
| --- | --- | --- | --- | --- | --- |
|  | Quitter  (N=67,843) | Reducer I  (N=27,987) | Reducer II  (N=47,628) | Sustainer  (N=162,645) | Increaser  (N=59,249) |
|  |  |  |  |  |  |
| Body mass index (kg/m^2^) |  |  |  |  |  |
| Pre | 24.9 ± 3.2 | 24.8 ± 3.4 | 25.0 ± 3.4 | 24.9 ± 3.4 | 25.0 ± 3.5 |
| Post | 25.0 ± 3.2 | 24.7 ± 3.3 | 24.8 ± 3.4 | 24.7 ± 3.3 | 24.8 ± 3.4 |
| Pre-post difference | 0.1 ± 1.5 | -0.2 ± 1.4 | -0.2 ± 1.3 | -0.2 ± 1.3 | -0.2 ± 1.3 |
| Waist circumference (cm) |  |  |  |  |  |
| Pre | 86.1 ± 8.4 | 86.2 ± 8.3 | 86.4 ± 9.0 | 86.2 ± 8.4 | 86.4 ± 9.1 |
| Post | 86.3 ± 8.0 | 85.9 ± 8.3 | 85.9 ± 8.3 | 85.8 ± 8.2 | 85.9 ± 8.4 |
| Pre-post difference | 0.2 ± 6.0 | -0.4 ± 5.4 | -0.4 ± 6.3 | -0.4 ± 5.5 | -0.4 ± 6.3 |
| Systolic blood pressure (mmHg) |  |  |  |  |  |
| Pre | 127.8 ± 15.0 | 128.2 ± 15.3 | 127.9 ± 14.8 | 127.9 ± 14.8 | 127.9 ± 14.8 |
| Post | 127.3 ± 14.6 | 127.0 ± 14.9 | 126.7 ± 14.4 | 126.7 ± 14.5 | 126.7 ± 14.7 |
| Pre-post difference | -0.5 ± 16.4 | -1.2 ± 16.6 | -1.2 ± 15.9 | -1.2 ± 15.8 | -1.2 ± 15.9 |
| Diastolic blood pressure (mmHg) |  |  |  |  |  |
| Pre | 79.4 ± 10.1 | 79.7 ± 10.3 | 79.9 ± 10.1 | 79.9 ± 10.1 | 79.9 ±10.1 |
| Post | 78.8 ± 9.8 | 78.8 ± 10.0 | 79.0 ± 9.9 | 79.0 ± 9.9 | 79.0 ± 10.0 |
| Pre-post difference | -0.7 ± 11.3 | -0.9 ± 11.4 | -0.8 ± 11.1 | -0.9 ± 11.0 | -0.9 ±11.2 |
| Fasting glucose (mg/dL) |  |  |  |  |  |
| Pre | 149.0 ± 47.3 | 152.1 ± 50.2 | 151.8 ± 48.5 | 151.5 ± 47.5 | 152.3 ± 49.1 |
| Post | 138.4 ± 50.8 | 137.7 ± 52.4 | 136.6 ± 50.8 | 137.4 ± 51.5 | 139.3 ± 54.1 |
| Pre-post difference | -10.6 ± 56.9 | -14.4 ± 58.4 | -15.2 ± 56.3 | -14.1 ± 55.6 | -13.1 ± 57.9 |
| Total cholesterol (mg/dL) |  |  |  |  |  |
| Pre | 198.3 ± 44.9 | 199.3 ± 44.0 | 200.5 ± 47.0 | 200.1 ± 44.8 | 199.3 ± 46.5 |
| Post | 192.0 ± 46.3 | 192.1 ± 43.7 | 193.5 ± 41.7 | 194.1 ± 42.9 | 193.7 ± 41.6 |
| Pre-post difference | -6.3 ± 49.4 | -7.2 ± 45.0 | -7.0 ± 45.5 | -6.1 ± 44.6 | -5.6 ± 45.0 |

Data are presented as mean ± standard deviation.

Pre-post difference, P value <0.001 for all.

^a^ Quitter, those who ceased smoking; Reducer I, those who reduced the number of cigarettes by 50% or more; Reducer II, those who reduced the number of cigarettes by 20% or more and by less than 50%; Sustainer, those who reduced the number of cigarettes by less than 20% or increased by less than 20%; Increaser, those who increased the number of cigarettes by 20% or more.
